# Gut microbiota as a therapeutic target of Chinese herbal medicine in allergic rhinitis: the gut–nasal axis and immunity

**DOI:** 10.3389/fphys.2026.1900200

**Published:** 2026-09-04

**Authors:** Ziye Huang, Na Sun

**Affiliations:** Guanghua Hospital Affiliated to Shanghai University of Traditional Chinese Medicine, Shanghai, China

**Keywords:** allergic rhinitis, Chinese herbal medicine, gut microbiota, gut-nasal axis, immunity

## Abstract

Allergic rhinitis (AR) is a prevalent chronic noninfectious inflammatory disorder. Emerging evidence suggests that the gut microbiota, as a central component of the human microecological system, plays an important role in the development and progression of AR through the gut–nasal axis. Chinese herbal medicine (CHM) has been shown to improve clinical symptoms and quality of life in patients with AR, and these therapeutic benefits may be related to its capacity to modulate gut microbial composition and activity. This review outlines the effects of CHM on the gut microbiota in AR and further explores the possible mechanisms involved.

## 1 Introduction

Allergic rhinitis (AR) is an immunoglobulin E (IgE)-mediated chronic inflammatory disease of the nasal mucosa. Its onset and progression are not solely driven by local allergic responses within the nasal mucosa, but are also shaped by systemic immune status, microbial ecological balance, and metabolic regulation (Zhang et al., 2026). With advances in microbiome research and mucosal immunology, the conceptual framework for AR has gradually expanded from localized nasal inflammation to a broader network of systemic immune regulation (Jude and Favour, 2025). In this context, the “gut–nasal axis” has emerged as a mechanistic framework that provides a new perspective for understanding the remote regulatory interactions between the gut microbiota and nasal mucosal immunity.

The gut–nasal axis refers to a bidirectional regulatory network through which the gut microbiota and the nasal cavity communicate via immune, metabolic, and neuroendocrine pathways (Huang et al., 2026; Qie et al., 2026). The gut, nasal cavity, and lung are developmentally related and share common features of the mucosal immune system (Lou et al., 2020). Through maintaining intestinal barrier integrity, modulating microbial metabolites, and shaping systemic immune responses, the gut microbiota may participate in the regulation of inflammatory processes in the nasal mucosa.

**Figure 1:**
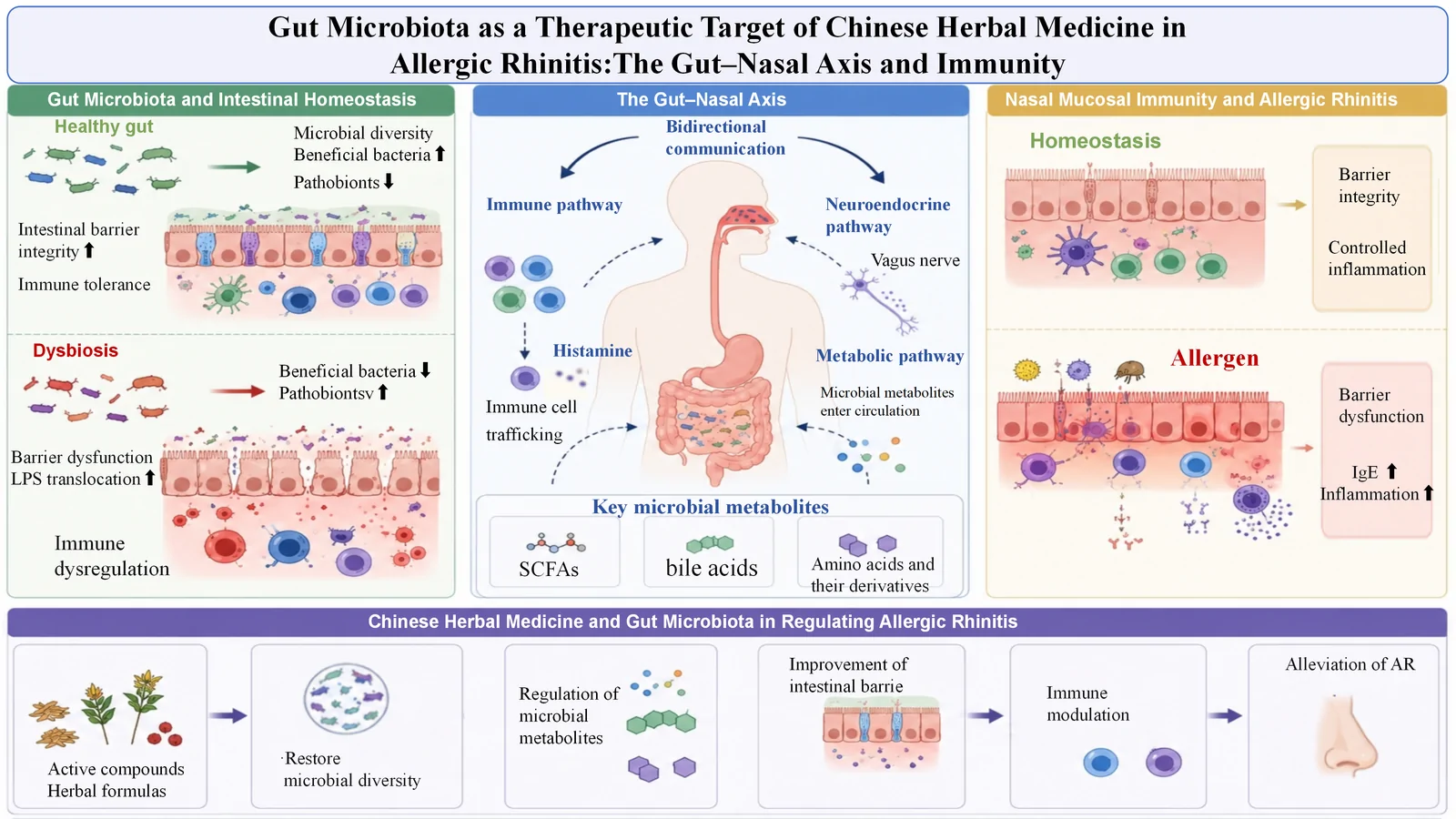
Article summary.

Traditional Chinese medicine emphasizes holistic regulation and considers the gut, lung, and nose to be closely interconnected in both physiological function and pathological processes. This concept broadly aligns with the modern gut–nasal axis, which emphasizes remote mucosal immune regulation across anatomically distinct sites. The novelty of this review lies in integrating Chinese herbal medicine (CHM), the gut microbiota, and immune regulation in AR into a unified mechanistic framework. In addition to systematically summarizing the bidirectional interactions between the gut microbiota and the onset and progression of AR, this review further explores the reciprocal interplay between the gut microbiota and bioactive constituents or compound formulas of CHM. By elucidating how the gut microbiota may serve as both a modulator of the therapeutic efficacy of CHM and a potential therapeutic target for AR, this review aims to provide a theoretical basis for mechanistic investigations and the development of personalized CHM-based interventions for AR (**Figure 1**).

## 2 Microbial exposure and gut dysbiosis in the susceptibility to AR

The development of AR is closely associated with insufficient early-life microbial exposure, gut microbiota dysbiosis, and impaired establishment of immune tolerance. The hygiene hypothesis proposes that limited microbial exposure during early life may disrupt normal immune maturation, resulting in enhanced T helper 2 (Th2)-skewed responses and impaired immune regulation, ultimately increasing susceptibility to allergic diseases (Perkin and Strachan, 2022). The microbiota hypothesis suggests that the diversity of gut microbiota plays a vital role in the development of host immunity, and that dysbiosis can lead to the emergence of allergic disorders (Noverr and Huffnagle, 2005).

Early life represents a critical window for the establishment of the gut microbiota and the maturation of the immune system. Microbial colonization begins during delivery, and maternal intestinal and vaginal microorganisms acquired through vaginal birth provide an important foundation for the initial establishment of the neonatal microbiota. After birth, breast milk serves as a major source of microorganisms and immunomodulatory factors, thereby facilitating gut microbial colonization and immune system development (Koren et al., 2024). In contrast, cesarean delivery may disrupt this natural colonization process. A meta-analysis of 22 studies involving 1,464,868 participants showed that cesarean delivery was associated with an increased risk of childhood AR (OR = 1.19, 95% CI: 1.12–1.27, P < 0.001). The association was more pronounced among children with a family history of allergy (OR = 1.82, 95% CI: 1.36–2.43) and those delivered by elective cesarean section (OR = 1.24, 95% CI: 1.05–1.46) (Liu et al., 2023). In addition, a study conducted in the United States suggested that exclusive breastfeeding may exert a preventive effect against AR (Rosas-SC et al., 2022). Excessive antibiotic exposure during early life may likewise reduce gut microbial diversity, disrupt host immune homeostasis, and consequently increase susceptibility to allergic diseases (Kesavelu and Jog, 2023).

Accumulating evidence indicates that alterations in the composition and function of the gut microbiota are closely associated with both the development and severity of AR. Animal studies have shown that gut microbiota dysbiosis can exacerbate respiratory allergic responses, whereas probiotic interventions may alleviate airway hyperresponsiveness and inflammation and suppress increases in IgE levels (Fiocchi et al., 2022). The gut microbial composition of children with AR also differs from that of healthy children. Dysregulation of specific bacterial subpopulations has been associated with IgE-mediated allergic responses and may increase susceptibility to AR during early childhood (Chiu et al., 2019). In children with house dust mite (HDM)-induced AR, the intestinal abundance of *Escherichia fergusonii* was reduced and negatively correlated with HDM-specific IgE levels. Further experiments demonstrated that oral supplementation with *E. fergusonii* attenuated allergic responses in mice (Li et al., 2025a). Collectively, these findings suggest that the gut microbiota participates in the immune regulation of AR and may represent a potential therapeutic target. Environmental factors, particularly air pollution, may also contribute indirectly to the development and progression of AR by disturbing the gut microbiota. Fouladi et al. (2020) reported increased abundances of intestinal *Firmicutes* and *Bacteroides* following exposure to air pollution. In an animal study, Liu et al. (2021) found that PM2.5 exposure reduced the abundance of *Bacteroidetes* in the murine gut and induced alterations in multiple bacterial genera. Further research revealed that changes in *Alistipes* and *Ileibacterium*, together with disrupted bile acid metabolism, were associated with aggravated nasal mucosal injury in PM2.5-exposed mice with AR (Li et al., 2025b). Although the respiratory tract is the primary site affected by airborne pollutants, these findings suggest that the gut microbiota may act as an important mediator linking environmental exposure to AR and provide evidence for cross-organ microbial regulation through the gut–nasal axis. In summary, the gut microbiota not only contributes to the development and progression of AR but may also serve as a mechanistic link between environmental exposure and allergic inflammation of the nasal mucosa. Therefore, therapeutic strategies targeting the gut microbiota may provide novel approaches for the prevention and treatment of nasal disorders, including air pollution-related AR.

## 3 Mechanisms by which the gut microbiota influences AR

### 3.1 Shared mucosal immunity and immune cell trafficking

Although the gut and nasal cavity are anatomically distinct, both constitute integral components of the mucosal immune system. The mucosal immune system is generally organized into immune induction sites and effector sites. Among these, mucosa-associated lymphoid tissue (MALT) represents a major immune inductive structure, with gut-associated lymphoid tissue (GALT) and nasopharynx-associated lymphoid tissue (NALT) serving as typical examples (Zhang et al., 2025). The lamina propria, as a key effector site, is enriched with immune cells such as effector T cells, plasma cells, macrophages, and dendritic cells, which collectively participate in antigen recognition, mucosal defense, and the maintenance of immune tolerance (Kim and Jang, 2020). In addition, NALT is an important mucosal immune induction site in the upper respiratory tract. It can initiate local protective immune responses against inhaled antigens and contribute to the regulation of mucosal immune homeostasis (Seefeld et al., 2024). Thus, GALT and NALT should not be viewed as isolated immune compartments; rather, they may be interconnected through the common mucosal immune system, forming cross-site immune communication.

Within this shared mucosal immune network, the gut microbiota may first regulate the activation and differentiation of immune cells within GALT. The gut microbiota and its metabolites can act on dendritic cells, T cells, B cells, macrophages, and innate lymphoid cells, thereby influencing the expansion and functional status of immune cells in Peyer’s patches and mesenteric lymph nodes (Ni et al., 2023). Under physiological conditions, the gut microbiota contributes to the establishment of a tolerogenic immune environment by promoting the differentiation of Treg and inducing the production of immunoregulatory mediators, such as interleukin-10 (IL-10) and transforming growth factor-β (TGF-β). These processes help restrain excessive Th2-type inflammatory responses (Traxinger et al., 2022). Conversely, microbial dysbiosis may impair this tolerogenic immune programming, enhance pro-inflammatory signaling, and lower the threshold of host responsiveness to allergen stimulation.

More importantly, the mucosal immune system is characterized by a shared homing property. After antigenic stimulation, immune cells generated at mucosal inductive sites can exit these sites, enter the lymphatic system and bloodstream, and subsequently migrate to distant mucosal effector tissues. This process is mediated by chemokine receptors and adhesion molecules that direct tissue-specific homing (Russell and Mestecky, 2022; Kwon et al., 2026). Through the circulation, the gut microbiota may remotely shape the nasal mucosal immune microenvironment by modulating metabolites, cytokines, and immune cell trafficking. Specifically, gut-derived γδT17 cells can migrate to the nasal mucosa, where integrin α4β7-mediated directional homing may influence local interleukin-17A levels. Aberrant activation of this migratory axis may promote inflammatory cell recruitment and epithelial inflammatory responses in the nasal mucosa (Zhao et al., 2025). Immune-cell trafficking, however, represents only one route through which the gut microbiota can influence nasal mucosal immunity. The activation, differentiation, and migration of these cells are also shaped by microbial products and inflammatory signals entering the systemic circulation. Under physiological conditions, the intestinal barrier restricts the translocation of such signals. When this barrier is disrupted, microbial components and metabolites may gain greater access to the circulation, amplify systemic inflammation, and further modify the activation and homing of immune cells to the nasal mucosa.

### 3.2 Intestinal barrier dysfunction and amplification of systemic inflammation

The intestinal barrier is a dynamic and selectively permeable interface that maintains host internal homeostasis. It allows the selective absorption of nutrients while preventing the abnormal translocation of harmful substances, microorganisms, and microbial metabolites into the internal milieu (Ethridge et al., 2021). Structurally and functionally, the intestinal barrier consists mainly of mechanical, immune, chemical, and biological components (Chen et al., 2025). Among these, the mechanical barrier plays a central role in regulating intestinal permeability and is primarily maintained by intestinal epithelial cells and tight junction structures, including claudins, occludin, and zonula occludens (ZO) proteins. These tight junction proteins are essential for preserving epithelial integrity and restricting the penetration of luminal microorganisms and toxins. .

**Figure 2:**
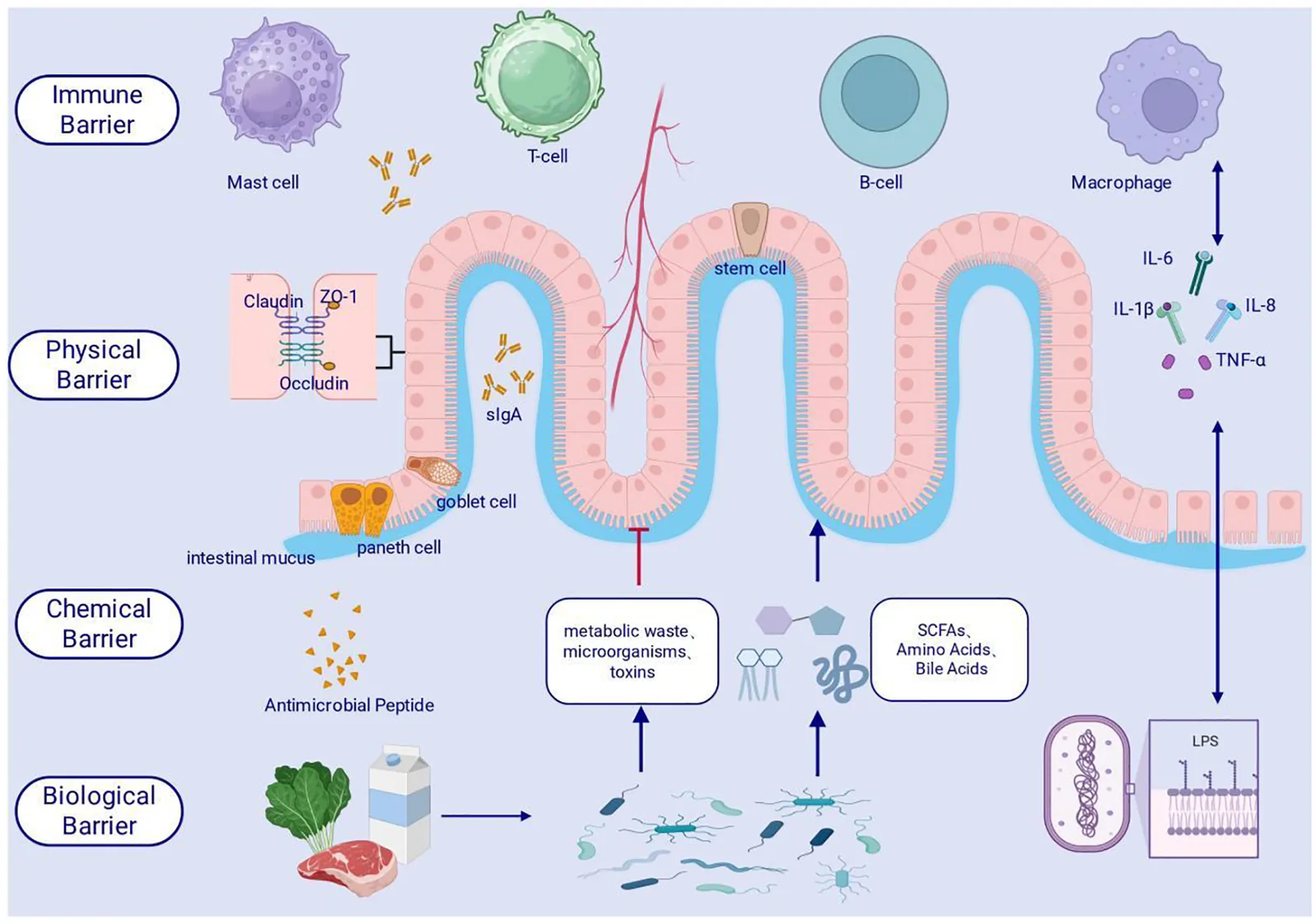
The intestinal barrier comprises immune, mechanical, chemical, and biological components. It exhibits permeability. Certain microbial metabolites aid in maintaining the intestinal barrier. LPS from Gram-negative bacteria penetrate the compromised barrier, stimulating immune cells to produce inflammatory mediators, which in turn exacerbate barrier damage.

Under conditions of gut microbial homeostasis, the microbiota and its metabolites promote the expression of tight junction proteins, maintain mucus layer stability, and support local immune homeostasis, thereby preserving intestinal epithelial barrier integrity. In contrast, gut microbiota dysbiosis may weaken barrier defense, increase intestinal permeability, and facilitate the entry of gut-derived signals, such as lipopolysaccharide (LPS), into the systemic circulation. Circulating LPS can activate Toll-like receptor 4 (TLR4) and trigger systemic inflammatory responses (Di Vincenzo et al., 2024; Almansour et al., 2025). Persistent inflammatory activation may further disrupt tight junction structures, thereby forming a self-amplifying cycle of dysbiosis, barrier disruption, and inflammatory propagation. In the development and progression of AR, intestinal barrier impairment may amplify nasal mucosal inflammation through systemic immune activation. Animal studies have shown that a high-fat diet reduces the expression of ZO-1 and occludin in colonic tissues of AR rats, increases serum LPS levels, and elevates inflammatory cytokines and ovalbumin(OVA)-specific IgE levels in both nasal lavage fluid and serum. These findings suggest that intestinal barrier disruption and LPS translocation may contribute to the amplification of AR-related inflammation (Peng et al., 2025). Evidence from fecal microbiota transplantation (FMT) studies further supports this mechanism from a reverse-intervention perspective. FMT can restore gut microbial homeostasis in mouse models of AR, ameliorate pathological injury in both colonic and nasal mucosal tissues, and regulate the balance of CD4+ T-cell subsets (Dong et al., 2024). Therefore, intestinal barrier dysfunction may participate in the onset and persistence of AR through endotoxin translocation, systemic inflammatory activation, amplification of nasal mucosal inflammation, and immune imbalance. This process represents one of the key mechanisms by which the gut–nasal axis mediates immune regulation in AR. (**Figure 2**).

### 3.3 Mechanisms of gut microbiota-derived metabolites in AR

Gut microbiota-derived metabolites serve as key molecular mediators linking alterations in the intestinal microbial ecosystem to immune responses in the nasal mucosa and constitute a critical component of long-range immunoregulatory signaling along the gut–nasal axis. Through the fermentation of dietary substrates and the biotransformation of host-derived nutrients, gut microorganisms generate a diverse array of bioactive metabolites, including short-chain fatty acids(SCFAs), bile acids(BAs), and amino acid derivatives, which subsequently contribute to the regulation of host immune homeostasis and inflammatory responses.

**Figure 3:**
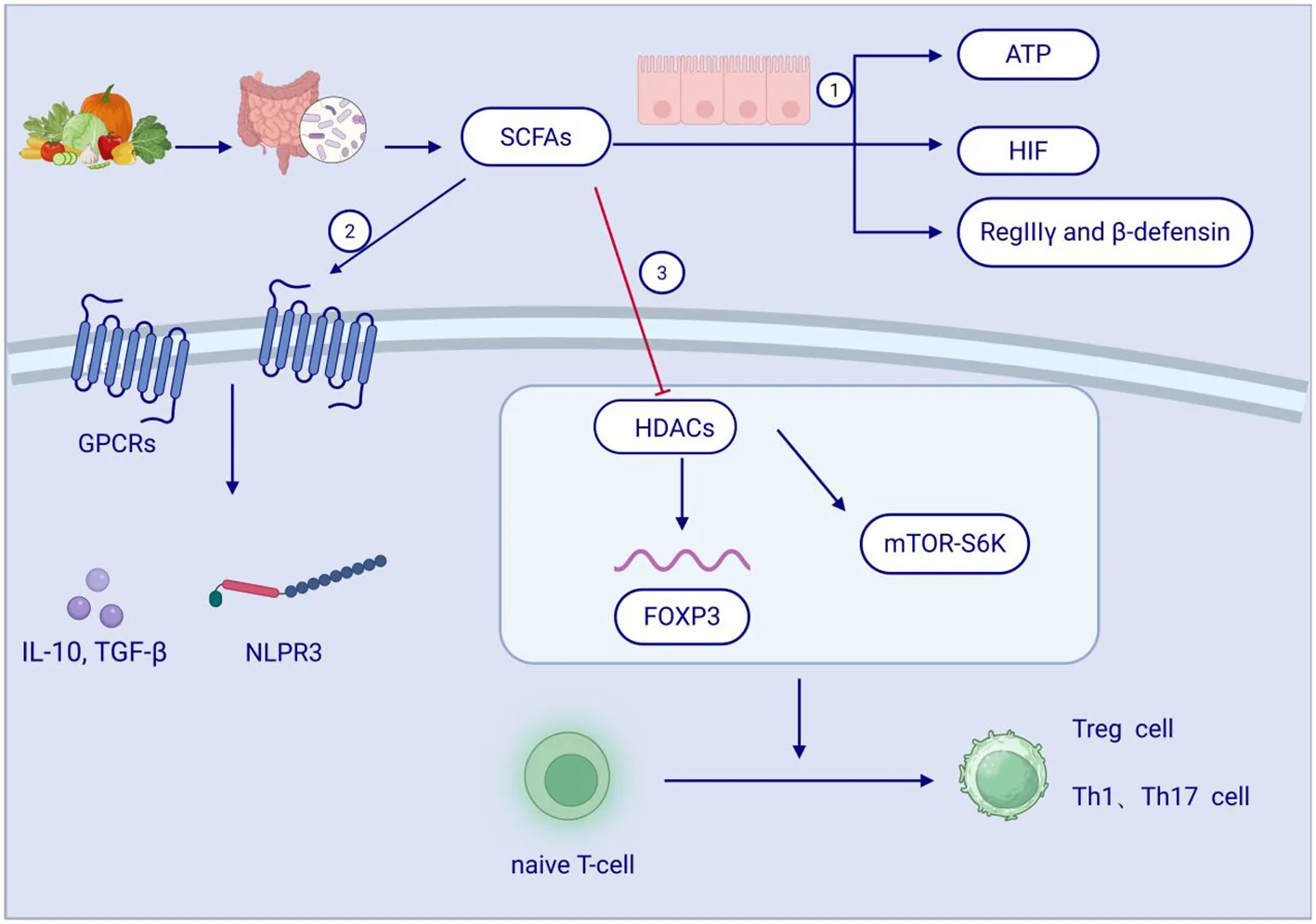
SCFAs provide energy and maintain the intestinal barrier. SCFAs activate GPCR signaling pathways. SCFAs inhibit HDACs to promote FOXP3 expression and activate the mTOR-S6K pathway.

SCFAs are a class of organic acids formed from the fermentation of dietary fiber and other indigestible carbohydrates in the gut, with acetate, propionate, and butyrate as the principal components. Their production is closely associated with bacterial genera such as *Bacteroides, Bifidobacterium*, and certain *Clostridium* species (Koh et al., 2016). At the phylum level, *Bacteroidetes* are important producers of acetate and propionate, whereas *Firmicutes* are closely related to butyrate synthesis (Den et al., 2013). Multiple clinical studies suggest that SCFAs levels are closely related to the risk of allergic diseases. Cohort studies have shown that lower fecal SCFAs levels in the first year of life are linked to a higher risk of subsequent allergy-related outcomes in children (Cheng et al., 2022). In addition, patients with AR show decreased abundance of Firmicutes and lower levels of SCFAs (Zhou M. et al., 2021), and AR mice with gut microbiota dysbiosis likewise show decreased SCFAs accompanied by aggravated symptoms (Chen Z. et al., 2022). Collectively, these findings indicate that SCFAs deficiency may represent an important link through which gut microbiota dysbiosis promotes the development and progression of AR. SCFAs may regulate disease development through multiple mechanisms. First, as an important energy source for colonic epithelial cells, SCFAs enhance cellular oxygen consumption and upregulate hypoxia-inducible factor expression, thereby maintaining an anaerobic luminal environment and preserving intestinal barrier integrity (Kelly et al., 2015). Second, butyrate stimulates intestinal epithelial cells to produce antimicrobial factors, including regenerating islet-derived protein IIIγand β-defensins. These molecules help shape the microbial community, particularly Gram-positive bacteria, and further reinforce intestinal barrier function (Zhao et al., 2018). In addition, SCFAs can enhance the expression of the transcription factor forkhead box P3 (FOXP3) through histone deacetylase (HDAC) inhibition and G protein-coupled receptor (GPCR) activation, thereby promoting the differentiation of naive T cells into Treg cells in the gut (Kaisar et al., 2017). Under specific conditions, SCFAs may also enhance mammalian target of rapamycin–ribosomal protein S6 kinase (mTOR–S6K) signaling through HDAC inhibition, thereby promoting the differentiation of T helper 1 (Th1) cells, T helper 17 (Th17) cells, and interleukin-10-positive (IL-10^+^) regulatory T cells (Park et al., 2015). Moreover, SCFAs can activate the NOD-like receptor family pyrin domain-containing 3 (NLRP3) inflammasome through GPCR-mediated signaling pathways, further contributing to the regulation of immune balance (Macia et al., 2015). Overall, the role of SCFAs in AR is mainly reflected in maintaining intestinal barrier homeostasis, regulating the microbial ecosystem, promoting immune tolerance, and limiting excessive inflammatory responses. (**Figure 3**).

BAs are major components of bile. Primary bile acids (PBAs) are synthesized in the liver from cholesterol and are conjugated with taurine or glycine before being secreted into bile. Upon entering the intestine, PBAs are further metabolized by the gut microbiota through dehydroxylation, epimerization, and oxidation to form secondary bile acids, such as deoxycholic acid and lithocholic acid. Beyond their classical role in lipid digestion and absorption, BAs also function as important signaling molecules that regulate host immune responses via receptors including the farnesoid X receptor, Takeda G protein-coupled receptor 5, and vitamin D receptor (Yang D. et al., 2025). Increasing findings suggest that dysregulated bile acid metabolism is closely associated with allergic airway diseases. Multi-omics analyses have demonstrated that mouse models of AR exhibit altered bile acid profiles, characterized by increased levels of taurochenodeoxycholate, deoxycholic acid, and cholic acid in serum, alongside reduced fecal concentrations of specific secondary bile acids (Chen et al., 2023). This pattern indicates a disruption of gut microbiota-mediated bile acid transformation, likely reflecting impaired conversion from primary to secondary bile acids and consequent imbalance in bile acid signaling. Under conditions of fine particulate matter (PM2.5) exposure, gut microbiota dysbiosis is accompanied by bile acid metabolic disturbances, which can activate the NLRP3 inflammasome pathway, leading to elevated interleukin-1β (IL-1β) levels and disruption of the nasal epithelial barrier. Notably, cholic acid levels have been positively correlated with IL-1β, suggesting that bile acids may contribute to AR exacerbation through inflammatory amplification (Li et al., 2025b). In addition, clinical observations of BAs metabolic abnormalities in patients with AR accompanied by constipation further support the role of BAs as key metabolic mediators within the gut–nasal axis (Wang C. et al., 2025). BAs-based interventions may alleviate allergic airway inflammation. For instance, chenodeoxycholic acid suppresses Th2-associated cytokines, including IL-4,IL-5, and IL-13, as well as TNF-α, thereby attenuating airway inflammation (Shaik et al., 2015). Similarly, tauroursodeoxycholic acid significantly alleviates HDM -induced allergic airway inflammation and improves airway hyperresponsiveness in mouse (Siddesha et al., 2016). However, current evidence is largely based on associative findings, and the causal roles of specific bile acid subtypes and their receptor-mediated signaling pathways in AR require further clarification. Furthermore, most interventional studies are limited to animal models, and robust clinical evidence regarding the efficacy and safety of bile acid-targeted therapies in patients with AR is still lacking.

Intestinal amino acids are derived from the hydrolysis of dietary proteins and are further transformed by the gut microbiota into a variety of bioactive metabolites, including amines, organic acids, and small-molecule derivatives. Amino acid-derived metabolites, particularly those related to tryptophan metabolism, are essential for maintaining intestinal barrier integrity, regulating mucosal immunity, and modulating allergic inflammation.

Tryptophan, the only amino acid containing an indole ring, can be metabolized into multiple bioactive compounds, including kynurenine, indole and 5-hydroxy tryptamine(5-HT) (Yang D. et al., 2025). Tryptophan-derived metabolites, such as indole-3-aldehyde and indole-3-propionic acid, regulate Th17/Treg immune balance through activation of the aryl hydrocarbon receptor(AhR). AhR signaling has been shown to suppress Th17-mediated inflammatory responses and attenuate disease severity (Calvo-Barreiro et al., 2023). In addition, indole and its derivatives can activate both AhR and the pregnane X receptor, thereby modulating intestinal mucosal immune cell function, limiting excessive inflammation, and maintaining intestinal barrier integrity. Notably, indole exhibits dual immunomodulatory effects, inhibiting Th17 cell differentiation while promoting Treg cell expansion (Ren et al., 2025). 5-HT, another key tryptophan metabolite, plays a significant signaling role in the gastrointestinal tract and is involved in modulating intestinal metabolic activity, mucosal homeostasis, and immune-inflammatory responses (Jiang et al., 2024). Furthermore, tryptophan can be converted into kynurenine via indoleamine 2,3-dioxygenase 1 (IDO1). Beyond its role in immunometabolism, IDO1 contributes to intestinal homeostasis by enhancing the differentiation of epithelial secretory cells and stimulating mucus production, which helps stabilize the intestinal microenvironment (Alvarado et al., 2019). In addition to tryptophan metabolism, other amino acids and their derivatives may also contribute to immune regulation in AR. For example, taurine has been reported to enhance interleukin-35 (IL-35) production, promote CD4^+^ T cell and Treg responses, and partially alleviate AR symptoms (Zhou J. et al., 2021). Moreover, in children with AR accompanied by constipation, gut microbiota dysbiosis has been linked to disturbances in aromatic amino acid metabolism. Alterations in taxa such as *Tyzzerella* may be linked to enhanced kynurenine metabolism and activation of Th17-related pathways, whereas *Bifidobacterium longum* has been shown to increase mucosal permeability and promote IgE release through the production of tyrosine-derived metabolites (Wang C. et al., 2025). Overall, these findings indicate that the gut microbiota contributes to AR pathogenesis by generating amino acid-derived metabolites with immunomodulatory properties, which act through receptor-mediated signaling, regulation of immune cell differentiation, and modulation of intestinal barrier function.

### 3.4 Neuroendocrine regulation via the vagus nerve

In addition to circulating immune cells and microbial metabolites, neuroendocrine signaling may represent another important route of communication along the gut–nasal axis. As a key component of the neuroimmune regulatory network within this axis, the vagus nerve may mediate the long-range effects of the gut microbiota and its metabolites on immune homeostasis in the nasal mucosa. The gut microbiota may suppress mast cell degranulation and histamine release in the nasal mucosa through vagus nerve-associated neuroimmune regulation. Meanwhile, microbiota-derived metabolites may modulate vagal activity and thereby influence respiratory immune responses (Yang W. et al., 2025). Moreover, vagal efferent signaling may further enhance anti-inflammatory effects by strengthening the intestinal epithelial barrier, reducing intestinal permeability, and reshaping the composition of the gut microbiota (Liu et al., 2025).

### 3.5 Histamine regulation by the gut microbiota in AR

Histamine is one of the key mediators linking the gut microbiota to AR. The gut microbiota can produce bacterially derived histamine through histidine decarboxylase (HDC), which enters the circulation, activates histamine receptors, and participates in systemic immune regulation. An EAACI position paper indicated that certain intestinal bacteria, such as *Lactobacillus* and *Escherichia*, are capable of synthesizing histamine, and that the H2 receptor plays an important role in regulating the Th2/Treg balance, suggesting that the “gut microbiota–histamine–immune” axis may influence susceptibility to allergic diseases (Jutel et al., 2023). Animal studies further demonstrated that the microbiota-derived metabolites can inhibit histamine release from mast cells in the lungs and nasal mucosa, thereby alleviating nasal mucosal inflammation and related symptoms (Folkerts et al., 2020). Oral administration of *Lacticaseibacillus paracasei* GOLDGUT-Lpc969 can improve the phenotype of allergic rhinitis by inhibiting mast cell degranulation and reducing histamine levels in serum and nasal cavity (Zhou et al., 2024).

## 4 Modulation of AR by herbal molecules and the gut microbiota

To compare the intestinal transformation characteristics of different types of bioactive molecules derived from CHM and their interactions with the gut microbiota, the available studies are categorized into four major groups: polysaccharides, polyphenols, saponins, and multi-component herbal active substances.

### 4.1 Polysaccharides

Current animal experimental evidence indicates that polysaccharides derived from different plant sources may interact with the gut microbiota and host tissues through distinct mechanisms. *Lonicera japonica* polysaccharide have been shown to upregulate the expression of β-catenin, E-cadherin, zonula occludens-1 (ZO-1), and occludin (Bai et al., 2022), whereas low-molecular-weight licorice polysaccharides increase goblet cell density and promote mucus secretion (Song et al., 2023). Polysaccharides derived from *Sanghuangporus* have been demonstrated to enrich beneficial microbial communities while reducing potentially pathogenic bacteria, thereby promoting short-chain fatty acid production and modulating immune responses (Wang J. et al., 2025).

Notably, the chemical structure of polysaccharides plays a critical role in determining their bioavailability and biological activity. Ginseng-derived residual oligosaccharides were more effective than ginseng polysaccharides in alleviating the symptoms of allergic rhinitis and were accompanied by marked alterations in gut microbial composition (Tao et al., 2025). This finding suggests that the gut microbiota exhibits substrate preferences for different glycan structures, with low-molecular-weight or more readily degradable polysaccharides being more efficiently utilized and exhibiting greater biological activity.

In addition, *Lycium barbarum* polysaccharide (LBP) can be further purified into *Lycium barbarum* glycopeptide (LbGP), which exhibits more pronounced biological activity than its parent polysaccharide. Studies have shown that LbGP enriches *Odoribacter* and *Duncaniella* while suppressing *Alistipes* and *Ruminococcus*. This microbial remodeling normalizes taurine and bile acid metabolism, restores the Th1/Th2 balance, and inhibits antigen-specific immunoglobulin E (IgE) synthesis, which ultimately drives the alleviation of allergic airway inflammation (Bai et al., 2025).

### 4.2 Polyphenols

Polyphenols are naturally occurring plant compounds that typically contain one or more hydroxyl-substituted aromatic rings. This structural feature confers strong redox capacity and efficient free radical scavenging ability, thereby endowing polyphenols with potent antioxidant, anti-inflammatory, and immunomodulatory properties (Farhan et al., 2024). In allergic diseases, the biological effects of polyphenols extend beyond their direct pharmacological activity and are closely linked to gut microbiota–mediated metabolic transformation. Polyphenols are abundant in commonly used CHM, including *Curcuma longa*, *Scutellaria baicalensis*, *Lonicera japonica*, and *Polygonum cuspidatum*. However, as typical xenobiotics, most native polyphenols exhibit low oral bioavailability and generally require enzymatic biotransformation by the gut microbiota to generate more absorbable and biologically active metabolites. Therefore, both inter-individual variability in the composition and metabolic activity of the gut microbiota, as well as the intrinsic chemical structure of polyphenols, jointly determine their metabolic conversion efficiency and ultimate pharmacological effects. Curcumin, a lipophilic polyphenol derived from the rhizome of *Curcuma longa*, serves as a representative example. It can be metabolized by various gut microbial species, among which *Escherichia coli* exhibits relatively strong metabolic activity. Through nicotinamide adenine dinucleotide phosphate(NADPH)-dependent curcumin/dihydrocurcumin reductases, curcumin is converted into metabolites such as dihydrocurcumin and tetrahydrocurcumin, which display improved stability and bioavailability (Scazzocchio et al., 2020). Notably, both curcumin and tetrahydrocurcumin reshaped the gut microbial community, including reducing the *Firmicutes*-to-*Bacteroidetes* ratio. Fecal microbiota transplantation from curcumin- or tetrahydrocurcumin-treated donors partially transferred the attenuation of Th2-mediated airway inflammation to recipient mice, with tetrahydrocurcumin-derived microbiota conferring greater protection than curcumin-derived microbiota. These findings demonstrate that microbial remodeling induced by either compound is sufficient to mediate at least part of its anti-allergic effect (Wu et al., 2021).

Flavonoids, an important subclass of polyphenols, face similar challenges of low bioavailability. Baicalin, a representative flavonoid glycoside, typically requires deglycosylation by β-glucuronidase produced by colonic microbiota to be converted into its aglycone form, baicalein, which exhibits enhanced lipophilicity and improved systemic absorption (Wang et al., 2024). Experimental evidence indicates that this biotransformation is markedly impaired under germ-free conditions, emphasizing the critical role of the gut microbiota in baicalin metabolism (Seradj et al., 2024). In OVA-induced AR models, baicalin has been reported to relieve allergic symptoms and regulate metabolic pathways, including histidine metabolism (Chen et al., 2019), while baicalein exerts anti-inflammatory effects through activation of nuclear receptor subfamily 4 group A member 1 and inhibition of the protein arginine methyltransferase 1/nuclear factor-κB (NF-κB) signaling pathway (Xu et al., 2025). Moreover, baicalin has additionally been observed to suppress allergy-associated inflammation via modulation of gut microbiota composition (Wang et al., 2022).

### 4.3 Saponins

Saponins are a group of plant-derived natural glycosides characterized by hydrophobic aglycones bound to hydrophilic sugar moieties via glycosidic linkages. Commonly found in CHM such as *Panax ginseng*, *Glycyrrhiza*, *Astragalus*, and *Panax notoginseng*, they exhibit both anti-inflammatory and anti-allergic activities. For instance, ginsenoside Rd and fermented red ginseng have been shown to ameliorate AR symptoms and restore gut microbiota dysbiosis. This restoration is marked by increased abundance of *Bacteroidetes* and *Actinobacteria* together with reduced abundance of *Firmicutes*, and concomitant regulation of immune cells and related cytokine expression (Kim et al., 2019). Glycyrrhizic acid, upon oral administration, relies on gut microbiota—particularly β-glucuronidase secreted by the *genus Lactobacillus*—to undergo hydrolytic conversion into glycyrrhetinic acid (GA) (Shi et al., 2025). The latter exhibits stronger membrane permeability and significantly enhanced biological activity. GA exerts anti-allergic effects through a triple mechanism: first, by modulating T helper cell differentiation and suppressing Th2 immune responses; second, by reducing the levels of OVA-specific antibodies produced by B cells; and third, by suppressing calcium channel-associated protein expression and inhibiting Ca²^+^ influx, thereby reducing mast cell degranulation and the release of inflammatory mediators (Han et al., 2017). These processes suggest that saponins not only rely on gut microbiota for activation but also further act on multiple key immune links in the development and progression of AR.

### 4.4 Multi-component herbal active substances

Beyond single compounds, multi-component herbal active substances also hold significant importance in the intervention of AR. Konjac-mulberry leaf compound powder is a functional plant-based preparation that combines konjac-derived glucomannan with flavonoids, alkaloids, and polysaccharides from mulberry leaves. This formulation promotes gut microbial fermentation, increases total cecal SCFAs, and corrects Th1/Th2 imbalance, thereby enhancing immune regulation (Zhang et al., 2023). *Dendrobium* extracts, which contain *Dendrobium* polysaccharides and phenolic compounds, can increase the abundance of microbial taxa associated with Treg induction while reducing taxa associated with OVA-sIgE induction (Duan et al., 2022).

By contrast, aqueous extracts of *Saposhnikovia divaricata* more clearly illustrate the multidimensional regulatory features of traditional wind-dispelling herbs. These extracts contain chromones (such as cimifugin), coumarin glycosides (such as 5-O-methylvisammioside), and a small amount of water-soluble polysaccharides. Studies suggest that they can remodel the gut microbiota in AR mice and regulate key pathways, including amino acid metabolism, polyamine synthesis, and energy metabolism, thereby contributing to the improvement of allergic symptoms (Chen Y. et al., 2022) (**Table 1**).

**Table 1:** Modulation of AR by herbal molecules and the gut microbiota.

CHM	Model	Gut microbiota	Metabolites/metabolic pathways	Immune modulation	Literature
*Lonicera japonica* polysaccharide	AR model established using LPS + IFN-γ- induced THP-1 cells	Maintained gut microbiota balance	repaired intestinal mechanical barrier	Inhibited NLRP3-driven inflammation and Th17 responses	(Bai et al., 2022)
low-molecular-weight *Glycyrrhiza* polysaccharide	Cyclophosphamide -induced mouse model	↑*Associated taxa included Muribaculaceae_unclassified* ↓ *Firmicutes and Proteobacteria*	↑SCFAs	Enhanced barrier and immune function, restored Th1/Th2 balance, and inhibited ERK/p38/NF-κB signaling	(Song et al., 2023)
*Sanghuangporus vaninii* polysaccharides	AR mouse	↑*Kineothrix, Lachnospiraceae bacterium UBA3282*, *Porcincola, Prevotella* sp. *CAG:873, Dysosmobacter, Roseburia* sp. *1XD42–69* **↓***Limosilactobacillus, Lactobacillus, Eubacterium-F, Intestinimonas*	-	Reduced nasal symptoms, IgE, eosinophils, and inflammatory cytokines; increased CD4+/CD8+ ratio and restored Th1/Th2 balance	(Wang J. et al., 2025)
GRO-N and GP-N	AR Rats	↑*Lactobacillaceae* ↓*Ruminococcaceae*	Associated with saccharide metabolic pathways; GRO-N involved FcϵRI and IL-17 signaling, whereas GP-N involved FcγR-mediated	Reduced nasal symptoms, goblet cells, and inflammatory cytokines; improved immune imbalance, with GRO-N showing superior efficacy	(Tao et al., 2025)
*Lycium barbarum* glycopeptide	Airway inflammation mouse	↑*Odoribacter, Duncaniella* ↓*Alistipes, Ruminococcus*	Taurine and hypotaurine metabolism, bile acid secretion, and pyrimidine metabolism	Corrected Th1/Th2 imbalance, decreased Th17 cells, and increased Treg cells	(Bai et al., 2025)
Curcumin, Tetrahydrocurcumin	Asthmatic mouse	↓ *Firmicutes/Bacteroidetes ratio,* *Proteobacteria, Intestinimonas,* *unidentified Ruminococcaceae,* *Lachnospiraceae*	-	Reduced symptoms and Th2-mediated inflammation	(Wu et al., 2021)
Baicalin	AR rats	-	Regulating linoleic acid metabolism, arachidonic acid metabolism, histidine metabolism, the citric acid cycle and other amino acid pathways.	Reduced IgE, histamine, IL-1β, IL-4, IL-6, and TNF-α, with overall anti-allergic and anti-inflammatory effects	(Chen et al., 2019)
Fermented red ginseng, ginsenoside Rd	AR mouse	↑ *Bacteroidetes and Actinobacteria* ↓ *Firmicutes*	-	Reduced nasal symptoms, IgE, IL-4, IL-5, IL-13, mast cells, eosinophils, and Th2 cells	(Kim et al., 2019)
Glycyrrhizic acid	-	-	-	Restored Th1/Th2 balance, reduced IL-4, OVA-specific IgE/IgG1, stabilized mast cells, decreased vascular permeability, and inhibited Orai1/STIM1/TRPC1-mediated Ca²^+^ influx	(Han et al., 2017)
Konjac-mulberry leaf compound	AR mouse	-	↑SCFAs	Reduced nasal symptoms, eosinophil infiltration, serum histamine, OVA-sIgE, and IL-4, and improved nasal mucosal histopathology	(Zhang et al., 2023)
*Dendrobium nobile* extract	AR mouse	Reversed gut microbiota dysbiosis at the phylum and genus levels	inhibition of the PI3K/AKT/mTOR pathway and increased Forkhead box protein expression	Reduced allergic symptoms and lung/nasal inflammation, regulated IL-2, IL-4, IL-6, IL-10, IL-17, IFN-γ and improved Treg differentiation	(Duan et al., 2022)
*Saposhnikovia divaricata* Aqueous Extracts	AR mouse	Adjusting *Cytophagales, Burkholderia, Alteromonadales, Lactococcus, Clostridiaceae*	Enriching the L-arginine degradation II, glycogen degradation II, superpathway of polyamine biosynthesis III, and isoprene biosynthesis II pathways	Reduced nasal symptoms, goblet/mast cells, IgE, histamine, LTB4, IL-4, IL-5, IL-6, IL-17,TNF-α,and inflammatory cells; increased IL-2, IL-10, IFN-γ	(Chen Y. et al., 2022)

↑ indicates an increase in abundance or level, whereas ↓ indicates a decrease in abundance or level, depending on the parameter shown.

## 5 Modulation of AR by herbal formulas via gut microbiota

Studies investigating CHM for AR predominantly explore their capacity to remodel gut microbial communities, shift microbial metabolic profiles, preserve intestinal barrier integrity, and modulate immune-inflammatory responses. Yupingfeng San, formulated with Astragali Radix, Saposhnikoviae Radix, and Atractylodis Macrocephalae Rhizoma, is a classic prescription widely applied for AR prevention and treatment. Polysaccharides extracted from Yupingfeng San are reported to lower the abundance of detrimental genera including *Streptococcus spp.* and *Enterococcus spp.*, alongside elevating intestinal ZO-1 and claudin expression in weaning Rex rabbits (Sun et al., 2016). Another classical formula, Xiaoqinglong Decoction (XQLD), has been demonstrated to alleviate gut dysbiosis. Murine AR model studies indicate that XQLD relieves nasal mucosal inflammation partially through restructuring gut microbial composition (Liu et al., 2024). Clinical data further reveals AR patients achieving sustained remission display marked shifts in characteristic gut genera such as *Dialister*, *Roseburia*, *Bacteroides*, and Prevotella_9. Such shifts tend to remodel core metabolic functions covering peroxisome proliferator-activated receptor signaling, peroxisomal activity, and the tricarboxylic acid cycle (Zhu et al., 2021). In AR rat models, Mahuang Fuzi Xixin Decoction elevates short-chain fatty acid concentrations, upregulates intestinal ZO-1, and restores balanced Th17/Treg ratios. These microbial and barrier-modulating effects contribute to lowered IgE and histamine concentrations, as well as relief of nasal allergic manifestations (Liang et al., 2020). In HDM-induced AR mice, Shi-Bi-Lin (SBL) reconstructs gut microbiota by tuning the abundance of multiple genera including *Candidatus Saccharimonas*, *Faecalibacterium*, *Dubosiella*, *Olsenella* and *Bifidobacterium*. SBL also reshapes microbiota-derived metabolites such as aspartic acid and succinic acid, which in turn helps mitigate nasal mucosal injury and limit inflammatory cell infiltration (Li et al., 2024).

## 6 Current challenges and future perspectives

Although existing studies suggest that traditional Chinese herbal medicines may ameliorate AR by modulating the gut–nasal axis, the available evidence remains subject to several important limitations. Most studies report parallel changes in the gut microbiota, microbial metabolites, and immune parameters but do not establish the microbiota as a necessary mediator. These microbial shifts may drive therapeutic effects or simply reflect reduced inflammation. The chemical complexity and variability of herbal medicines across sources, processing methods, extraction procedures, and doses also compromise reproducibility. Gut microbial biotransformation may further generate metabolites with distinct activities or toxicities, making it difficult to disentangle the effects of parent compounds, specific taxa, and microbial metabolites. Most mechanistic evidence derives from murine models, whereas clinical studies are often limited by small sample sizes, short follow-up, inadequate placebo controls, and insufficient adjustment for diet, concomitant medications, and environmental exposures. Moreover, the therapeutic effects of herbal medicines should not be attributed solely to the gut microbiota, which likely represents only one component of their broader multicomponent and multitarget pharmacological network.

Future studies should combine antibiotic-mediated microbiota depletion, fecal microbiota transplantation, receptor-knockout models, and microbiome multi-omics to distinguish causal microbial alterations from secondary changes. Multi-omics approaches should also trace the *in vivo* biotransformation of traditional Chinese medicine constituents and clarify the interactions among parent compounds, specific microbial strains, and microbial metabolites. Clinically, standardized, randomized, double-blind, placebo-controlled multicenter trials are warranted, with longitudinal monitoring of the gut microbiota, metabolites, and immune parameters. Artificial intelligence and machine-learning methods may integrate microbiome, metabolome, clinical phenotype, and phytochemical data to identify biomarkers of treatment response, recurrence, and adverse events and to develop predictive models. Precision microbiome profiling may further define allergic rhinitis subtypes at the strain and functional-pathway levels. Integrating these subtypes with age, diet, allergen type, disease severity, and traditional Chinese medicine syndrome patterns may support personalized formula selection and optimization of dose and treatment duration.

## 7 Conclusion

This review outlines the important role of the gut microbiota in AR pathogenesis and highlights its therapeutic potential. From the perspective of the gut–nasal axis, the gut microbiota affects the onset and progression of AR through multiple interconnected mechanisms, including regulation of immune homeostasis, maintenance of intestinal barrier integrity, and mediation of metabolite-driven signaling pathways. CHM, as a multi-target and holistic therapeutic approach, demonstrates distinct advantages in modulating gut microbiota composition, functional activity, and host immune responses. These properties provide a novel conceptual framework and promising option for AR prevention and treatment.
